# High-coverage targeted lipidomics revealed dramatic lipid compositional changes in asthenozoospermic spermatozoa and inverse correlation of ganglioside GM3 with sperm motility

**DOI:** 10.1186/s12958-021-00792-3

**Published:** 2021-07-07

**Authors:** Shuqiang Chen, Ming Wang, Li Li, Jun Wang, Xuhui Ma, Hengde Zhang, Yang Cai, Bin Kang, Jianlei Huang, Bo Li

**Affiliations:** Department of Obstetrics and Gynecology, Tangdu Hospital, the Fourth Military Medical University, 710038 Xi’an, China

**Keywords:** Asthenozoospermia, Lipidomics, Sperm motility, GM3

## Abstract

**Background:**

It has been previously demonstrated that cholesterol content and cholesterol/phospholipid ratio were significantly higher in asthenozoospermia and oligoasthenoteratozoospermia. The majority of published studies have investigated the fatty acid composition of phospholipids rather than lipids themselves. This study evaluated the lipid composition of asthenozoospermic and normozoospermic spermatozoa, and identified the exact lipid species that correlated with sperm motility.

**Methods:**

A total of 12 infertile asthenozoospermia patients and 12 normozoospermia subjects with normal sperm motility values were tested for semen volume, sperm concentration, count, motility, vitality and morphology. High-coverage targeted lipidomics with 25 individual lipid classes was performed to analyze the sperm lipid components and establish the exact lipid species that correlated with sperm motility.

**Results:**

A total of 25 individual lipid classes and 479 lipid molecular species were identified and quantified. Asthenozoospermic spermatozoa showed an increase in the level of four lipid classes, including Cho, PE, LPI and GM3. A total of 48 lipid molecular species were significantly altered between normozoospermic and asthenozoospermic spermatozoa. Furthermore, the levels of total GM3 and six GM3 molecular species, which were altered in normozoospermic spermatozoa versus asthenozoospermic spermatozoa, were inversely correlated with sperm progressive and total motility.

**Conclusions:**

Several unique lipid classes and lipid molecular species were significantly altered between asthenozoospermic and normozoospermic spermatozoa, revealing new possibilities for further mechanistic pursuits and highlighting the development needs of culture medium formulations to improve sperm motility.

**Supplementary Information:**

The online version contains supplementary material available at 10.1186/s12958-021-00792-3.

## Introduction

The plasma membrane is an essential structural component of living cells, which serves as a specialized barrier separating the cell interior from the external environment and enabling intracellular organelles to be compartmentalized [1, 2]. The plasma membrane lipids play vital roles in a variety of physiological and pathological processes by sustaining the structure and function of organelles [1, 3]. Plasma membrane lipids contain four main classes: glycerophospholipids, sphingolipids, glycolipids and sterols [4]. These lipids exhibit critical biological functions, including energy storage, membrane structure construction, cell signaling and transcriptional regulation [5]. Drastically altered levels of distinct lipid species were correlated with cellular processes [2, 4].

The fluidity and flexibility of cell membranes are mainly dependent on their lipid constitution [6]. Integrity and fluidity of sperm membranes are important for sperm motility, capacitation, acrosome reaction (AR), and sperm-egg fusion [7]. Sperm membrane components, particularly lipids, are directly involved in core sperm functions, including membrane fluidity, sperm motility and spermatozoon-oocytecross-talk [6, 8]. The sperm plasma membrane lipids are roughly made up of 70 % phospholipids, 25 % neutral lipids, and 5 % glycolipids [9]. The fatty acid composition of spermatozoaplasma membrane is directly related to sperm motility [10, 11]. Cholesterol is important for modulating the membrane fluidity, and its levels in the sperm membrane of infertile patients are dramatically higher than those in fertile men [12].

Approximately 15 % of couples are affected by infertility globally, and 50 % of infertile couples are due to male-factor infertility [13]. Asthenospermia, defined as total sperm motility < 40 % and progressive motility < 32 % in a semen sample, is one of the common causes of male infertility, affecting approximately 40 % of all cases [14, 15]. It was found that spermatozoal stearic acid was negatively correlated, and DHA was positively correlated with sperm motility [16].The concentration of phosphatidylcholine (PC) and phosphatidylethanolamine (PE) of sperm membrane were reduced in asthenozoospermic samples [17]. However, the sperm lipid composition of normospermic men and lipid changes in asthenozoospermic semen samples have not been fully explored. Advances in lipidomics are enabling detailed understanding of lipids in biological membranes [18–20]. Lipidomics is the study of the entire lipid pool within a cell, tissue, or organism, which provides a quantitative analysis of the lipid profile in that sample [21]. Lipidomics has already been used to elucidate the fatty acid groups or classes in spermatozoa from different species [22]. However, quantifying the levels or amounts of lipids has proven to be more difficult than detecting their presence. This is a rather difficult task when the cells number is limited and the quantification of the lipids needs to be set to a higher threshold level than the current one [23]. With technological advancements in MS, recent efforts have been made to design a streamlined method to fractionate and quantify the lipids in sperm cells via HPLC/MS-based approaches [24].

Understating the lipids related to sperm motility is necessary to help the researchers to address the subject of reduced sperm motility and produce an accurate diagnosis of infertility [6]. The purpose of this study was to investigate the lipid composition of male sperm in normozoospermic spermatozoa and asthenozoospermic semen samples in order to better elucidate the role of lipids on male fertility. In the precent study, Exion UPLC coupled with a SCIEX QTRAP 6500 PLUS system was first used for the study of lipidomics in human spermatozoa. The targeted library tailored for human sperm lipidome conferred sufficient lipid coverage to render global lipid pathway analysis [25].

## Materials and methods

### Ethical approval

 This study was approved by the Internal Review and Ethics Boards of Tangdu Hospital, and written informed consent was obtained from all participants at enrollment. This study was conducted in accordance with the Declaration of Helsinki [26].

### Study population

The present study was performed at the Reproductive Unit of the Department of Obstetrics, Tangdu Hospital of the Fourth Military Medical University from January 2018 to August 2019. All participants had no diagnosed diseases related to mitochondrial dysfunction as well as no history of smoking and alcohol intake. Semen samples were collected from 12 healthy men with normal sperm concentration, total sperm count, and total and progressive mobility, as well as 12 asthenozoospermia patients who masturbated after three days of abstinence in the course of infertility work-up. We excluded participants with azoospermia, chromosome alterations, anti-sperm antibodies, undescended testis, varicocele, history of epididymitis, orchitis, epididymo-orchitis, human papillomavirus infection and/or sexually transmitted infections because of their proved negative influence on fertility and semen parameters according to previous reports [27–29]. The protocol was approved by the Internal Review and Ethics Boards of Tangdu Hospital, and written informed consent was obtained from all healthy males and patients.

### Sample preparation

After liquefaction, each semen sample was analyzed according to the World Health Organization criteria (WHO, 2010). Patients were excluded upon identification of any significant androgenic or endocrine abnormalities. The patients found to have chromosomal aberrations by karyotype testing were also excluded. Samples with abnormal pH and viscosity, increased number of leukocytes or immature germ cells, and abnormal semen liquefaction were excluded. Asthenozoospermia was defined as the progressive motility of sperm < 32 % within 60 min of ejaculation. The fresh human semen specimens were centrifuged using a two-layer gradient system including an upper layer solution of 40 % Percoll gradient and a lower layer solution of 80 % Percoll gradient (GE Healthcare, Waukesha, WI, USA) (400 g, 15 min) to separate spermatozoa from seminal plasma. Spermatozoa were then washed twice with 2.0 ml PBS at 300 g for 5 min. Each sample was diluted to 1.0 ml with PBS, and then the sperms count were calculated by a hemocytometer as previously described [30]. Subsequently, 100 µL of sperm solution was prepared at a concentration of 1 × 10^8^ cells/ml for lipid extraction.

### Lipid extraction

Lipids were extracted from the sperm samples using a modified version of the Bligh and Dyer’s method as previously described [31]. Briefly, 750 µl of ice-cold chloroform: methanol (1:2, v/v) with 10 % deionized H_2_O was added to inactivate the samples. Then samples were homogenized on an automated bead ruptor (Omni, USA) and further centrifuged at 1500 rpm and 4 °C for 1 h. Next, 350 µl of deionized H_2_O and 250 µl of chloroform were added to generate separate phase. After transferring the lower organic phase into another tube, 500 µl of chloroform was added for a second extraction. The two extractions were pooled and dried using SpeedVac (Genevac, UK). The dried samples were stored at − 80 °C for mass spectrometric analysis.

### Targeted lipidomics

#### Analysis of polar lipids

The detailed analytical protocol was described previously [31, 32]. Briefly, the individual lipid class of polar lipids were separated by normal phase (NP)-HPLC using a Phenomenex Luna 3 μm silica column (i.d.150 × 2.0 mm) under the following conditions: mobile phase A (chloroform:methanol:ammonium hydroxide, 89.5:10:0.5) and mobile phase B (chloroform:methanol:ammonium hydroxide:water, 55:39:0.5:5.5), the flow rate was 270 µl/min, and column oven temperature was 25 °C. The gradient started with 5 % of B and was held for 3 min, then increased to 40 % of B over 9 min, and held at 40 % for 4 min before further increasing to 70 % of B over 5 min. The gradient was maintained at 70 % of B for 15 min before returning to 5 % of B over 3 min, and was finally equilibrated for 6 min. Individual polar lipid species were quantified by referencing the spiked internal standards including PC-14:0/14:0, d31-PC16:0/18:1, d31-PE-16:0/18:1, d31-PS-16:0/18:1, PS-17:0/20:4, PA-17:0/17:0, PA-17:0/20:4, PG-14:0/14:0, d31-PG-16:0/18:1, GluCer-d18:1/8:0, Cer-d18:1/17:0, C14:0-LBPA, d31-PI-16:0/18:1, S1P-d17:1, Sph-d17:1, SM-d18:1/12:0, LPC-17:0, LPE-17:0, LPI-17:0, LPA-17:0, LPS-17:0 obtained from Avanti Polar Lipids (Alabaster, AL, USA) and PI-8:0/8:0 from Echelon Biosciences, Inc. (Salt Lake City, UT, USA). GM3 species were quantified using GM3 d18:1/17:0 synthesized in-house as an internal standard.

#### Analysis of neutral lipids

Neutral lipids (TAGs, DAGs and CEs) were analyzed using a modified version of reverse-phase HPLC/MRM as previously described [33, 34]. Briefly, the lipids were separated on a Phenomenex Kinetex 2.6 μm C18 column (i.d. 4.6 × 100 mm^2^) using an isocratic mobile phase of chloroform:methanol:0.1 M ammonium acetate (100:100:4) at a flow rate of 170 µl/min for 17 min. Levels of short, medium, and long-chain TAGs were calculated by referencing the spiked internal standards of TAG(14:0)3-d5, TAG(16:0)3-d5 and TAG(18:0)3-d5 obtained from CDN isotopes, respectively. DAGs were quantified using d5-DAG16:0/16:0 and d5-DAG18:1/18:1 (Avanti Polar Lipids) as internal standards. Free cholesterols and cholesteryl esters were analyzed as previously described with d6-cholesterol and d6-C18:0 cholesteryl ester (CE) (CDN isotopes) as internal standards.

#### Analysis of free cholesterol

Free cholesterols and total cholesteryl esters were analyzed using HPLC/APCI/MS/MS as previously described, with d6-Cho and d6-CE-18:1 (CDN isotopes) as internal standards [31].

### Statistical analyses

The statistical differences between the normozoospermic and asthenozoospermic sample were determined using Student’s *t*-test or Wilcoxon rank-sum test with Shapiro-Wilk Normality test. Correlations were assessed by Pearson’s correlation coefficient (r). All analyses were performed using SPSS. Results were considered to be statistically significant if *p* < 0.05. For all analyses, *p represented *p* < 0.05, **p represented *p* < 0.01.

## Results

### Patients andsemen samples

A total of 12 patients with asthenozoospermia and 12 healthy fertile men were included in this study. The mean age was 33.1 years for asthenozoospermia patients, and 32.4 years for healthy fertile men. There was no significant difference between the two groups. The parameters of the semen samples were listed in Table 1. The progressive and average sperm motility of asthenozoospermia patients was 26.42 ± 4.17 % and 48.42 ± 11.46 %, respectively, which were significantly lower than those of healthy fertile men (*p* < 0.0001 and *p* < 0.0001, respectively). There was no difference in sperm concentration, ejaculate volume and healthy sperm morphology between the two groups.

**Table 1:** The parameters of the semen samples

Parameters studied	Normozoospermia	Asthenozoospermia	*P* value
**Age of patients**	32.417 ± 4.716	33.083 ± 3.752	0.7172
**Sexual abstinence (days)**	3.667 ± 1.374	4.833 ± 1.280	0.0514
**pH**	7.383 ± 0.055	7.400 ± 0.000	0.3283
**Total sperm number (× 10^6)**	560.70 ± 278.23	465.22 ± 195.67	0.3620
**Ejaculate volume** (ml)	4.500 ± 1.256	3.979 ± 1.469	0.4238
**Sperm concentration** (× 10^6/ml)	133.000 ± 73.389	120.917 ± 44.000	0.6441
**Progressive motility (A + B)** (%)	53.700 ± 8.224	26.417 ± 4.166	< 0.0001 **
**Total motility**	70.750 ± 10.762	48.421 ± 11.457	0.0001 **
**Sperm vitality** (%)	84.167 ± 5.928	68.625 ± 8.070	< 0.0001 **
**Good sperm morphology** (%)	6.500 ± 2.021	5.375 ± 1.570	0.1589

Data are presented as Means±SD.

Significant at *P < 0.05; **P < 0.01 (by t test)

### The levels of individual lipid class in normozoospermic and asthenozoospermic spermatozoa

**Fig. 1:**
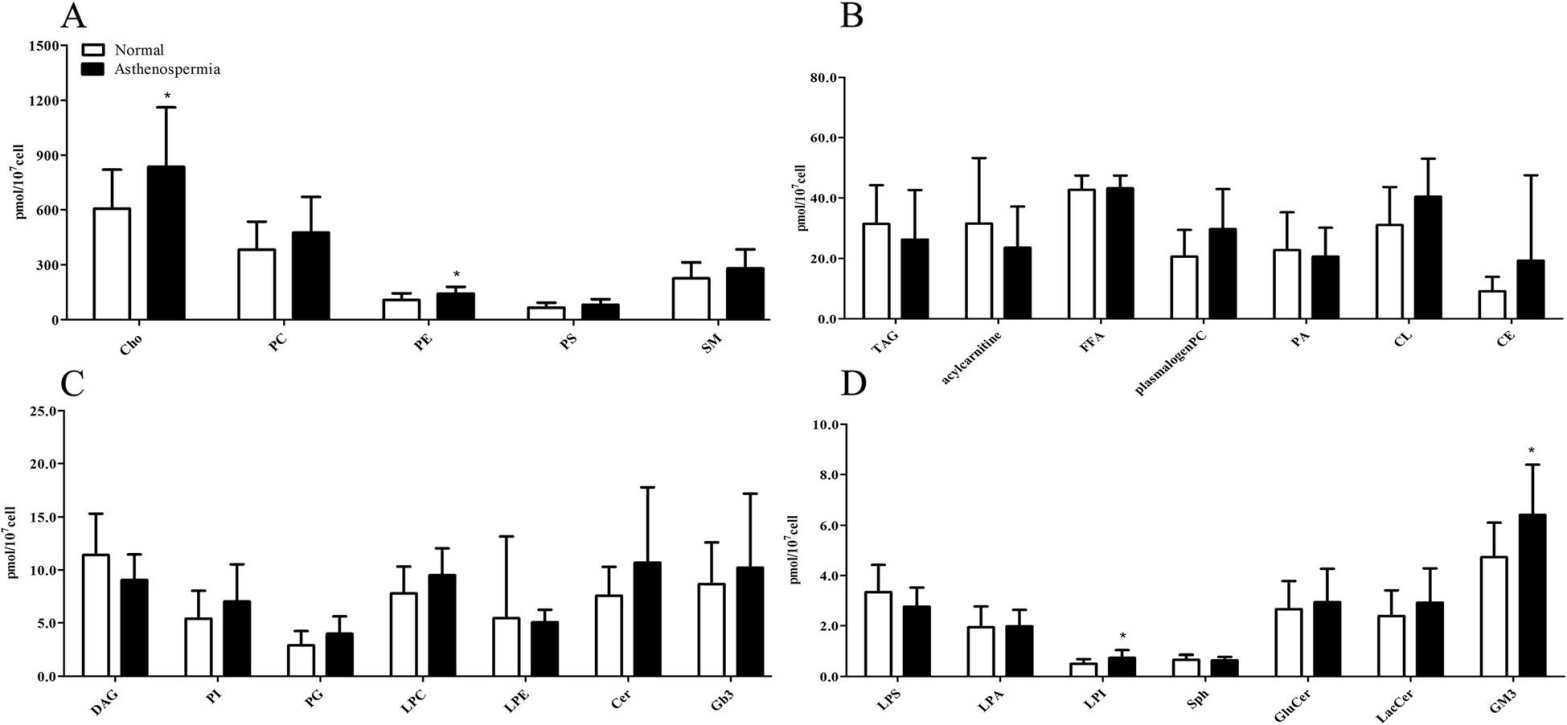
The levels of 25 individual lipid classes in normozoospermic and asthenozoospermic spermatozoa. (**A**) Changes in the levels of cholesterol (Cho) and phosphatidylethanolamines (PE)between the two groups examined are illustrated. (**B**) The levels of triacylglycerols (TAG), free fatty acids (FFA), phosphatidic acids (PA), cardiolipins (CL) and cholesteryl esters (CE)etc. (**C**) The levels of diacylglycerols (DAG), phosphatidylinositols (PI), phosphatidylglycerols (PG), and ceramide trihexoside (Gb3) etc. (**D**) Changes in the levels of lysophosphatidylinositols (LPI) and monosialodihexosyl gangliosides (GM3) between the two groups examined are illustrated.Significant at *P < 0.05. Error bars show standard deviation

A total of 25 lipid classes were identified and quantified in normozoospermic and asthenozoospermic spermatozoa (Fig. 1, Supplementary table-1), which included cholesterol (Cho), cholesteryl esters (CE), triacylglycerols (TAG), diacylglycerols (DAG), free fatty acids (FFA), phosphatidylcholines (PC), phosphatidylethanolamines (PE), phosphatidylinositols (PI), phosphatidylglycerols (PG), phosphatidylserines (PS), phosphatidic acids (PA), lysophosphatidylcholine (LPC), lysophosphatidylethanolamine (LPE), lysophosphatidylserine (LPS), lysophosphatidic acid (LPA), lysophosphatidylinositols (LPI), sphingomyelins (SM), ceramides (Cer), glucosylceramides (GluCer), lactosylceramides (LacCer), monosialodihexosyl gangliosides (GM3), sphingosine, (Sph), acylcarnitine, cardiolipins (CL) and ceramide trihexoside (Gb3).

The analyses of the total lipid fraction of individual lipid class were shown in Fig. 1 and Supplementary table-1. Surprisingly, contrary to normal lipid composition observed in human cells, Cho was the most abundant lipid species in normozoospermic and asthenozoospermic spermatozoa, followed by PC, SM, PE, PS, FFA, CL and TAG. Cho, PE and LPI in asthenozoospermic spermatozoa were significantly higher than those in normal controls (both p < 0.05) (Fig. 1). The level of GM3 in asthenozoospermic spermatozoa was significantly increased compared to normozoospermic spermatozoa, whereas plasmalogen PC, CL, PG and LPC showed a similar but insignificant trend (Fig. 1). The levels of DAG and LPS were lower in asthenozoospermic spermatozoa than those in the normal controls, but the difference was not significant (Fig. 1).

### Significantly altered lipid molecular species between normozoospermic and asthenozoospermic spermatozoa

**Fig. 2:**
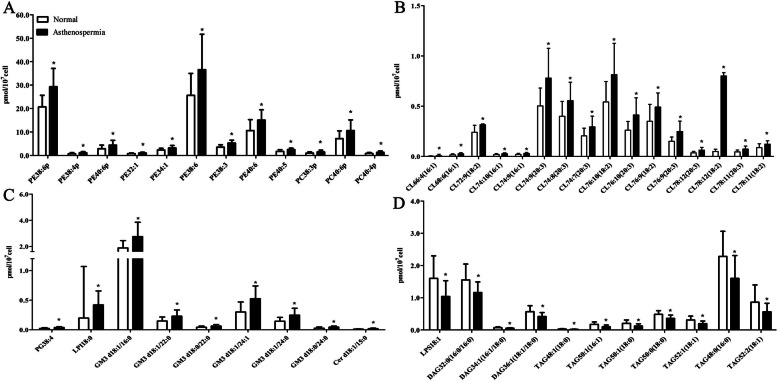
Significantly altered lipid molecular species between normozoospermic and asthenozoospermic spermatozoa. (**A**) Significantly altered phosphatidylethanolamines (PE) and phosphatidylcholines (PC) molecular species between normozoospermic and asthenozoospermic spermatozoa. (**B**) Significantly altered cardiolipins (CL) molecular species between normozoospermic and asthenozoospermic spermatozoa. (**C**) Significantly altered phosphatidylglycerols (PG), lysophosphatidylinositols (LPI), monosialodihexosyl gangliosides (GM3) and ceramides (Cer) molecular species between normozoospermic and asthenozoospermic spermatozoa. (**D**) Significantly altered lysophosphatidylserine (LPS), diacylglycerols (DAG) andtriacylglycerols (TAG) molecular species between normozoospermic and asthenozoospermic spermatozoa. Significant at *P < 0.05. Error bars show standard deviation

A total of 479 lipid molecular species were identified and quantified in the study, of which 48 species significantly changed between normozoospermic and asthenozoospermic spermatozoa (*p* < 0.05) (Fig. 2, Supplementary table-2). The altered lipid molecules belonged to PE, PC, PG, CL, LPI, LPS, Cer, GM3, DAG and TAG. PE plays a major role in the structure and function of mitochondrial membranes. In this study, the concentrations of nine PE molecules in asthenozoospermic spermatozoa were higher than those in control spermatozoa (*p* < 0.05) (Fig. 2). CL is a universal component of mitochondria in all eukaryotes, which is localized to the inner mitochondrial membrane (IMM) and plays an important role in the structural organization and the function of mitochondrial membranes [35, 36]. In this study, 55 CL molecular species were detected, and the concentrations of 16 CL molecules in asthenozoospermic spermatozoa were significantly increased compared to the control group (*p* < 0.05) (Fig. 2). GM3, the main ganglioside in majority of extraneural tissues of vertebrates, is one of the essential components of plasma membrane rafts [37]. Twenty GM3 molecular species were identified, and the concentrations of six GM3 molecules in asthenozoospermic spermatozoa were significantly increased compared to the control group (*p* < 0.05) (Fig. 2). TGs stored in lipid droplets (LDs) are energy substrates for β-oxidation, and precursors for membrane lipids and signaling molecules. The concentrations of seven TG molecules in asthenozoospermic spermatozoa were significantly decreased compared to the control group (*p* < 0.05) (Fig. 2).

### The levels of GM3 species were inversely correlated with sperm progressive motility

Furthermore, we aimed to explore the relationship between lipids levels and sperm progressive motility. The levels of total GM3 and LPI were negatively associated with sperm progressive motility (*p* < 0.05) (Table 2). The correlation of 48 significantly altered lipid species with sperm progressive motility was analyzed between normozoospermic and asthenozoospermic spermatozoa. The six significantly changed GM3 molecular species in normozoospermic spermatozoa versus asthenozoospermic spermatozoa included GM3 d18:1/16:0, GM3 d18:0/22:0, GM3 d18:0/24:0, GM3 d18:1/22:0, GM3 d18:1/24:0 and GM3 d18:1/24:1, which were inversely correlated with sperm progressive motility (*p* < 0.05) (Table 2). The levels ofLPI18:0, PE32:1, PE34:1 and PE38:6p were also negatively correlated with sperm progressive motility (*p* < 0.05) (Table 2). Interestingly, the only lipid that was positively correlated with sperm progressive motility was TAG52:1(18:1) (*p* < 0.05) (Table 2).

**Table 2:** Correlation of the lipids in spermatozoa with progressive motility (*n* = 24)

Lipids	Pearson correlation coefficient	*P* value
Total GM3	-0.443	0.03
Total LPI	-0.406	0.049
GM3 d18:1/16:0	-0.451	0.027
GM3 d18:0/22:0	-0.481	0.017
GM3 d18:0/24:0	-0.479	0.019
GM3 d18:1/22:0	-0.408	0.048
GM3 d18:1/24:0	-0.424	0.039
GM3 d18:1/24:1	-0.527	0.008
LPI18:0	-0.467	0.022
PE32:1	-0.426	0.038
PE34:1	-0.419	0.042
PE38:6p	-0.416	0.043
TAG52:1(18:1)	0.412	0.045

Correlations of lipids with progressive motility were tested by Pearson’s correlation analysis.Results were considered to be statistically significant if *P* < 0.05

### The levels of GM3 species were negatively correlated with sperm total motility

We further explored the relationship between lipids levels and sperm total motility. The concentrations of total Cho, GM3, LPI, PE and plasmalogen PC were negatively correlated with sperm total motility (*p* < 0.05) (Table 3). The correlation of 48 lipid molecular species with sperm total motility was also analyzed between normozoospermic and asthenozoospermic spermatozoa. The levels of five GM3 molecular species significantly changed between normozoospermic and asthenozoospermic spermatozoa, including GM3 d18:1/16:0, GM3 d18:0/24:0, GM3 d18:1/22:0, GM3 d18:1/24:0 and GM3 d18:1/24:1, which were negatively correlated with sperm progressive motility (*p* < 0.05) (Table 3). The levels of six significantly changed PE molecular species between normozoospermic and asthenozoospermic spermatozoa included PE32:1, PE38:4p, PE38:6p, PE38:6, PE40:6p and PE40:6, which were negatively correlated with sperm progressive motility (*p* < 0.05) (Table 3). The levels of LPI18:0, PC40:4p and PG38:4 were also negatively correlated with sperm progressive motility (*p* < 0.05) (Table 3).

**Table 3:** Correlation of the lipids in spermatozoa with total motility (*n* = 24)

Lipids	Pearson correlation coefficient	*P* value
Total Cho	-0.489	0.015
Total GM3	-0.533	0.007
Total LPI	-0.551	0.005
Total PE	-0.523	0.009
Total plasmalogenPC	-0.434	0.034
GM3 d18:1/16:0	-0.536	0.007
GM3 d18:0/24:0	-0.509	0.011
GM3 d18:1/22:0	-0.455	0.026
GM3 d18:1/24:0	-0.492	0.015
GM3 d18:1/24:1	-0.586	0.003
LPI18:0	-0.543	0.006
PC40:4p	-0.444	0.030
PE32:1	-0.434	0.034
PE38:4p	-0.524	0.009
PE38:6p	-0.649	0.001
PE38:6	-0.459	0.024
PE40:6p	-0.484	0.017
PE40:6	-0.493	0.014
PG38:4	-0.565	0.004

Correlations of lipids with progressive motility were tested by Pearson’s correlation analysis. Results were considered to be statistically significant if P < 0.05

## Discussion

In the present study, we presented the first detailed overview of the lipid composition of human sperm from normozoospermic spermatozoa samples. A total of 25 individual lipid classes, 479 lipid molecular species were identified and quantified by high-coverage targeted lipidomics. We also evaluated the differences in lipid components between normozoospermic andasthenozoospermic spermatozoa. The results showed that lipid alterations associated with asthenospermia included increased levels of cholesterol (Cho), PEs, LPIs and monosialodihexosyl GM3s. Furthermore, the levels of total GM3s were negatively correlated with sperm progressive motility and total motility. The widespread lipidomic shifts identified herein could partially explain the low sperm motility. This study presented a useful and quantitative lipidomic data repository of asthenozoospermic sperms to facilitate further mechanistic pursuits.

Spermatozoa membrane contains a heterogeneous mixture of phospholipids, glycolipids and sterols. Changes in the composition of the sperm plasma membrane phospholipids and fatty acids may culminate in membrane fluidity alteration, which may lead to impaired motility [6]. Lipidomics is a relatively new discipline that emerged in 2003. It essentially relies on the principles of analytical chemistry, which primarily centralized on mass spectrometry [20]. The fatty acid content of sperm cells has been a topic of investigation for several years [6]. Lipidomics has already been used to elucidate fatty acid groups or classes in spermatozoa from different species [22].The fatty acids lipidome of seminal fluid varied markedly between thenormozoospermia, oligoasthenoteratozoospermia, asthenozoospermia and varicocoele [16]. It has been found that the composition of saturated and unsaturated fatty acids in sperm is related to its motility, and asthenozoospermic semen samples showed lower levels of polyunsaturated fatty acids and higher levels of saturated fatty acids compared to normozoospermic samples [17]. A positive correlation between the levels of very long chain polyunsaturated fatty acids with sperm count and total motility count has been revealed by gas chromatography [38]. A significant higher cholesterol sulfate/seminolipid ratio in semen of oligoasthenozoospermic patients was observed compared with normal motility values [39].

Lipids are critical regulators of mammalian sperm function, enabling the process of capacitation, triggering acrosome exocytosis and ultimate fertilization [40]. However, the majority of published studies have investigated fatty acid composition of phospholipids, but have not achieved a satisfactory lipidomic coverage [6, 10, 38]. Since the selected reaction monitoring method is utilized in targeted lipidomics, it has provided high sensitivity for quantitative lipid analyses. With the advancement of technology, it is possible to detect and quantify low abundance lipids in human sperms with high sensitivity. In the present study, Exion UPLC coupled with a SCIEX QTRAP 6500 PLUS system was first used for the study of lipidomics in human spermatozoa. Through high-coverage targeted lipidomics, 25 individual lipid classes were detected and analyzed. Cho, PE, LPI and GM3 in asthenozoospermic spermatozoa were significantly higher those in the healthy group. It has been previously demonstrated that cholesterol content and cholesterol/phospholipid ratio were significantly higher in asthenozoospermia and oligoasthenoteratozoospermia [12, 41]. In accordance with the previous studies, higher cholesterol content was found in asthenozoospermicspermatozoa in this study. Little is known about the relationship between sperm phospholipid composition and male infertility. This study found that the levels of total PE and LPI were significantly increased in asthenozoospermic spermatozoa compared to the healthy group. Gangliosides, including GM1, GM3 and GD1a/GDlb, constitute a large family of sialic acid-containing glycosphingolipids, which play a key regulatory role in diverse cellular processes [42]. GM1 localization is highly conserved in the spermatozoa of diverse mammalian species, and changes in GM1 localization patterns in human sperms correspond to male fertility [43]. It has been shown that ganglioside GM1 regulate sperm acrosome exocytosis (AE) and fertilization competence through CaV2.3, and its activity has been highly controversial in sperms [40]. Fertilization is characterized by the triggering of long-lasting calcium (Ca^2+^) oscillations in the egg cytoplasm. It was noted that intracellular Ca^2+^ levels were increased, accompanied with a higher concentration of GT1b in a dose-dependent manner during oocyte IVM [44]. Accumulating data also showed that gangliosides were involved in regulation of cell proliferation, and GM1 could inhibit EGFR activation and promote contact inhibition of growth through regulating the distribution of EGFR from GEM domain to caveolae domain [45]. GM3 was thought to be involved in the modulation of various biological processes including cell proliferation and differentiation, apoptosis, embryogenesis, oncogenesis, etc. [37, 46]. Cell lines with high GM3 content showed decreased cell mobility, invasiveness and metastasis [37, 46]. GM3 could inhibit cell proliferation by perturbing the expression of factors modifying the cell cycle [37, 46]. In particular, GM3s appear to be the major ganglioside in the male reproductive system, and several studies have demonstrated their abundance in bovine, ovine, and human sperms [47–50]. GM3s are enriched in membrane rafts and localized to equatorial segment in mouse sperms [47]. GM3s are important component of lipid rafts, and their movements make sperms competent for fertilization [51]. GM3 might play a key role in spermatozoa maturation and fertility [52]. Although, due to technical reasons, we did not detect other gangliosides except GM3, it was found that the total GM3s levels in asthenozoospermic spermatozoa were significantly increased compared to the healthy group. Since GM3 was involved in transmembrane signaling modulation of growth factor receptor activities, as well as cell adhesion and motility, it was supposed that the increase of GM3 level might be an important reason for the decline of sperm motility in asthenozoospermic spermatozoa. Among the 479 lipid molecular species that were detected and analyzed, 48 lipid molecules were found to be significantly different between normozoospermic and asthenozoospermic spermatozoa. These 48 altered lipids belonged to 10 lipid classes, including PE, PC, PG, LPI, LPS, Cer, GM3, CL, DAG and TAG. Interestingly, it was found that among these altered lipids between the two groups, the glycerophospholipids and sphingolipids were significantly increased, but the glycolipids were significantly down-regulated in asthenozoospermic spermatozoa. These results suggested that increased phospholipids and reduced glycerolipids in spermatozoa were associated with impaired sperm motility.

The exact lipid species that correlate with sperm motility have not been conclusively established. The present study demonstrated that amongall 25 individual lipid classes that were detected and analyzed, there was a strong negative correlation between the total GM3s and LPI levels and sperm progressive motility. Among the altered molecular species between normozoospermic and asthenozoospermic spermatozoa, 10 lipids were negatively correlated with sperm progressive motility. Interestingly, five GM3 molecular species were negatively correlated with sperm progressive motility. Many studies have demonstrated that GM3s bind to the extracellular domain of epidermal growth factor receptor (EGFR), disrupting its dimerization and autophosphorylation, and inhibiting the tyrosine kinase activity of EGFR [52]. Exposure of COCs to exogenous GM3 could suppress meiotic maturation and restrict the expansion of cumulus cells during meiotic maturation via EGFR mediated PI3K/AKT signaling pathways, and could initiate apoptosis at high concentrations [53]. Epidermal growth factor (EGF) is one of the important cytokines that plays a role in spermatogenesis, spermatozoa epididymal maturation and capacitation. EGF can increase sperm concentration by stimulating the meiotic phase of spermatogenesis [54, 55], and stimulate human sperm capacitation by activating the tyrosine kinase of the EGFR [56]. EGF can also affect sperm motility parameters depending on its concentration and exposure time [57]. In asthenozoospermic spermatozoa, increased GM3s levels may inhibit the EGFR activity, which leads to decreased sperm motility. Incubation of human sperms with micelles made from glycerophospholipid mixtures increased sperm motility, which could be a relevant approach for the treatment of infertility in males [58]. It has also been found that EGF added to chilled ram sperms at concentrations of 200 ng·ml^− 1^ could significantly increase sperm total motility and progressive movement [57]. Herein, we hypothesized that supplementation of 200 ng·ml^− 1^EGF in the fertilization culture medium and then incubating the sperm at 37℃ under 5 % CO_2_ might improve sperm motility and enhance reproductive effects.

It should be noted that this study had some limitations. Firstly, we used a small sample comprising of 12 asthenozoospermia and 12 normozoospermia participants to elucidate the differences in lipids composition between the normozoospermic and asthenozoospermic spermatozoa, further validation from an independent and larger cohort of patients was needed. Based on the current study design, it was thus not possible to establish a direct association between the lipidomic changes and asthenozoospermia. In addition, the dysregulation of GM3s enrichment observed in asthenozoospermic spermatozoa awaited further mechanistic validation, although our preliminary evidence suggested that the increases in GM3s might be associated with low sperm motility. Finally, in order to obtain pure sperms, we used gradient centrifugation technology that could not separate seminal plasma at the same time. Therefore, we did not compare the differences in lipids composition between the normozoospermic and asthenozoospermic seminal plasma at the same time. In the future, we needed to compare the seminal plasma lipid composition between normozoospermia and asthenozoospermia patients. This would help reveal the reasons for the elevated GM3s levels in asthenozoospermic sperms, and establish a method to rapidly evaluate sperm quality by detecting the lipids content of seminal plasma.

## Conclusions

In conclusion, our work presented the first detailed overview of the lipid composition of the human sperm through high-coverage targeted lipidomics. By comparing the differences in the contents of lipids between normozoospermic and asthenozoospermic spermatozoa, we found that several unique lipid classes and lipid molecular species were linked to sperm motility, providing new possibilities for further mechanistic pursuits. To our knowledge, this was the first study to establish a correlation between the motility of human sperm and GM3 level.

## Supplementary Information

**Additional file 1.**

**Additional file 2.**

## Data Availability

The datasets used or analyzed during the current study are available from the corresponding author on reasonable request.

## Abbreviations

AR: Acrosomereaction
Cho: cholesterols
PC: phosphatidylcholines
PE: Phosphatidylethanolamines
PS: Phosphatidylserines
PI: Phosphatidylinositols
PG: Phosphatidylglycerols
PA: Phosphatidic acids
LBPA: Lyso-bisphosphatidic acids
LPC: Lysophosphatidylcholine
LPI: Lysophosphatidylinositols
CL: Cardiolipins
SM: Sphingomyelins
Cer: Ceramides
GluCer: Glucosylceramides
GM3: Monosialo-dihexosylgangliosides
DAG: Diacylglycerol
TAG: Triacylglycerols
CE: Cholesteryl esters
