# Supplementary material for: High-coverage targeted lipidomics revealed dramatic lipid compositional changes in asthenozoospermic spermatozoa and inverse correlation of ganglioside GM3 with sperm motility
Source: Reprod Biol Endocrinol. 2021 Jul 7;19:105. doi: 10.1186/s12958-021-00792-3 (PMC8262046; doi:10.1186/s12958-021-00792-3)
Supplement: Supplementary file 1 — Additional file 1. [file 12958_2021_792_MOESM1_ESM.doc]

**Supplementary table-1. The levels of individual lipid classes in spermatozoon**

| Main class | Normal  (pmol/10^7^cell) | Asthenospermia  (pmol/10^7^cell) | P value |
| --- | --- | --- | --- |
| Cho | 606±214 | 835±326 | 0.024* |
| PC | 384±153 | 477±194 | 0.203 |
| PE | 108±36 | 143±37 | 0.028* |
| PS | 66.2±27.0 | 80.9±30.9 | 0.229 |
| SM | 226±88 | 281±104 | 0.173 |
| TAG | 31.5±12.7 | 26.3±16.3 | 0.394 |
| acylcarnitine | 31.6±21.6 | 23.6±13.5 | 0.145 |
| FFA | 42.7±4.7 | 43.2±4.2 | 0.774 |
| plasmalogenPC | 20.6±8.9 | 29.8±13.1 | 0.057 |
| PA | 22.8±12.4 | 20.6±9.6 | 0.629 |
| CL | 31.1±12.5 | 40.4±12.6 | 0.083 |
| DAG | 11.4±3.9 | 9.05±2.41 | 0.090 |
| CE | 9.12±4.76 | 19.3±28.2 | 0.115 |
| PI | 5.39±2.64 | 7.02±3.5 | 0.210 |
| PG | 2.89±1.34 | 3.97±1.63 | 0.092 |
| LPC | 7.78±2.54 | 9.49±2.54 | 0.116 |
| LPE | 5.43±7.72 | 5.05±1.19 | 0.661 |
| LPS | 3.34±1.08 | 2.76±0.76 | 0.142 |
| LPA | 1.94±0.83 | 1.98±0.65 | 0.868 |
| LPI | 0.508±0.176 | 0.739±0.29 | 0.028* |
| Cer | 7.57±2.72 | 10.7±7.06 | 0.08 |
| Sph | 0.66±0.191 | 0.638±0.137 | 0.746 |
| GluCer | 2.66±1.12 | 2.94±1.33 | 0.582 |
| LacCer | 2.39±1.02 | 2.92±1.36 | 0.293 |
| Gb3 | 8.65±3.93 | 10.2±6.95 | 0.520 |
| GM3 | 4.73±1.37 | 6.41±1.98 | 0.030* |

mean ± SD, *P<0.05 was considered to be significant.
