# Supplementary material for: High-coverage targeted lipidomics revealed dramatic lipid compositional changes in asthenozoospermic spermatozoa and inverse correlation of ganglioside GM3 with sperm motility
Source: Reprod Biol Endocrinol. 2021 Jul 7;19:105. doi: 10.1186/s12958-021-00792-3 (PMC8262046; doi:10.1186/s12958-021-00792-3)
Supplement: Supplementary file 2 — Additional file 2. [file 12958_2021_792_MOESM2_ESM.doc]

**Supplementary table-2. The levels of individual lipid classes in spermatozoon**

| Main class | Normal  (pmol/10^7^cell) | Asthenospermia  (pmol/10^7^cell) | P value |
| --- | --- | --- | --- |
| PE38:6p | 20.7±4.95 | 29.3±7.8 | 0.002 |
| PE38:4p | 0.7±0.481 | 1.12±0.51 | 0.02 |
| PE40:6p | 2.87±1.54 | 4.40±2.03 | 0.02 |
| PE32:1 | 0.799±0.262 | 1.074±0.282 | 0.01 |
| PE34:1 | 2.29±0.77 | 3.18±1.06 | 0.01 |
| PE38:6 | 25.6±9.4 | 36.6±15.1 | 0.02 |
| PE38:3 | 3.52±1.03 | 5.26±1.25 | 0.001 |
| PE40:6 | 10.6±4.7 | 15.1±4.4 | 0.01 |
| PE40:5 | 1.73±0.75 | 2.49±0.62 | 0.006 |
| PG38:4 | 0.0227±0.0138 | 0.0375±0.015 | 0.01 |
| PC38:3p | 0.989±0.498 | 1.489±0.731 | 0.03 |
| PC40:6p | 7.197±3.285 | 10.604±4.537 | 0.02 |
| PC40:4p | 0.869±0.356 | 1.308±0.579 | 0.02 |
| LPI18:0 | 0.198±0.87 | 0.42±0.236 | 0.003 |
| LPS18:1 | 1.6±0.7 | 1.04±0.49 | 0.02 |
| GM3 d18:1/16:0 | 1.88±0.57 | 2.76±1.09 | 0.01 |
| GM3 d18:1/22:0 | 0.148±0.068 | 0.229±0.105 | 0.02 |
| GM3 d18:0/22:0 | 0.0436±0.0197 | 0.0633±0.0241 | 0.02 |
| GM3 d18:1/24:1 | 0.299±0.170 | 0.525±0.215 | 0.005 |
| GM3 d18:1/24:0 | 0.144±0.065 | 0.246±0.117 | 0.008 |
| GM3 d18:0/24:0 | 0.0269±0.0208 | 0.045±0.0181 | 0.02 |
| CL66:4(16:1) | 0.00321±0.00286 | 0.00641±0.01485 | 0.049 |
| CL68:6(16:1) | 0.0162±0.0087 | 0.0264±0.011 | 0.01 |
| CL72:9(18:2) | 0.24±0.0699 | 0.315±0.0089 | 0.02 |
| CL74:10(16:1) | 0.0181±0.0101 | 0.0263±0.0091 | 0.02 |
| CL74:9(16:1) | 0.0198±0.0112 | 0.0298±0.0093 | 0.01 |
| CL74:9(20:3) | 0.503±0.177 | 0.78±0.296 | 0.006 |
| CL74:8(20:3) | 0.4±0.148 | 0.555±0.183 | 0.02 |
| CL74:7(20:3) | 0.204±0.077 | 0.294±0.107 | 0.01 |
| CL76:10(18:2) | 0.542±0.204 | 0.814±0.311 | 0.009 |
| CL76:10(20:3) | 0.261±0.088 | 0.413±0.17 | 0.006 |
| CL76:9(18:2) | 0.35±0.168 | 0.491±0.141 | 0.02 |
| CL76:9(20:3) | 0.151±0.042 | 0.248±0.103 | 0.003 |
| CL78:12(20:3) | 0.036±0.0156 | 0.0628±0.0281 | 0.004 |
| CL78:12(18:2) | 0.0501±0.0218 | 0.801±0.0316 | 0.006 |
| CL78:11(20:3) | 0.0448±0.0195 | 0.0741±0.0286 | 0.004 |
| CL78:11(18:2) | 0.0875±0.0387 | 0.122±0.033 | 0.015 |
| Cer d18:1/15:0 | 0.011±0.0039 | 0.0182±0.0112 | 0.002 |
| DAG32:0(16:0/16:0) | 1.552±0.493 | 1.165±0.326 | 0.02 |
| DAG34:1(16:1/18:0) | 0.0723±0.0224 | 0.0559±0.0137 | 0.02 |
| DAG36:1(18:1/18:0) | 0.570±0.184 | 0.419±0.121 | 0.01 |
| TAG48:1(18:0) | 0.0283±0.0119 | 0.0191±0.009 | 0.02 |
| TAG50:1(16:1) | 0.173±0.0791 | 0.0994±0.0542 | 0.007 |
| TAG50:1(18:0) | 0.205±0.104 | 0.121±0.071 | 0.015 |
| TAG50:0(18:0) | 0.484±0.114 | 0.359±0.104 | 0.005 |
| TAG52:1(18:1) | 0.31±0.12 | 0.194±0.084 | 0.006 |
| TAG48:0(16:0) | 2.283±0.778 | 1.601±0.708 | 0.02 |
| TAG52:2(18:1) | 0.863±0.536 | 0.562±0.265 | 0.047 |

mean ± SD, *P<0.05 was considered to be significant.
